# Antimalarial activity of solvent fractions of a leaf of *Eucalyptus globulus labill* against *Plasmodium berghei* infected mice

**DOI:** 10.1186/s12906-022-03702-1

**Published:** 2022-08-16

**Authors:** Mihret Ayalew, Seyfe Asrade Atnafie, Azmeraw Bekele

**Affiliations:** 1 Department of Pharmacology, Institute of Health, Jimma University, Jimma, Ethiopia; 2 Department of Pharmacology, College of Medical and Health Science, University of Gondar, Gondar, Ethiopia; 3 Department of Social and Administrative Pharmacy, Institute of Health, Jimma University, Jimma, Ethiopia

**Keywords:** *Eucalyptus globulus* Labill, Leaf, Solvent fractions, Antimalarial, Mice. *Plasmodium berghei*

## Abstract

**Introduction:**

The leaf of *Eucalyptus globulus* is commonly used in the traditional management of malaria. However, the efficacy of solvent fractions are didn’t study yet scientifically. Thus, this study aimed to investigate the antimalarial efficacy of the solvent fractions of the leaf of *Eucalyptus globulus* in mice against *P.berghei*.

**Methods:**

The antimalarial activity of the fractions was tested in a 4-day suppressive test, Rane’s test, and prophylactic test models within P.berghei infected mice. The results were analyzed using a one-way analysis of variance (ANOVA) followed by a post hoc Tukey’s test in version 20 SPSS.

**Results:**

All fractions at all test doses in the three test models suppressed parasitemia (*p* < 0.001) compared to the negative controls. In addition, the CF and EA at all three test doses and the AF at 400 mg/kg in three antimalarial test models showed 50% and above parasitemia suppression. In compliance with this, all fractions at all test doses in all test models prolonged the mean survival time of the mice greater than 12 days, except the AF at a lower dose. All fractions at 400 mg/kg in the three test models prevented (*p* < 0.001) loss of body weight and rectal temperature compared to the negative controls. Furthermore, all fractions in all test models and doses prevented packed cell volume reduction (*p* < 0.05 to *p* < 0.001) compared to the negative controls..

**Conclusion:**

The findings of this study showed that CF and EAF had greater antimalarial activity compared to AF. This could be attributed to the presence of few phytochemicals in the AF in contrast to the CF and EAF. Overall, the results of this study further support the in vitro antimalarial activity study and the traditional use of the leaf in the management of malaria.

## Introduction

Malaria is a febrile infectious disease caused by plasmodium parasites including Plasmodium *falciparum, Plasmodiumvivax, Plasmodiummalariae, Plasmodiumovale,* and *Plasmodium. knowlesi.* From these, *P. falciparum* and *P. vivax* are the most common human malaria causes and *P. falciparum* is the most deadly parasite. The plasmodium parasites are spread to people through the bite of the female anopheles mosquito, called the malaria vector, which mainly bites at night [1]. Malaria infection could be either mild, manifested by chills, sweating, headache, muscular ache, malaise, anorexia, and /or nausea, or severe characterized by impaired consciousness, delirium or coma, respiratory distress, circulatory collapse, abnormal bleeding, acute renal failure, hemoglobinuria [2].

Despite malaria being both preventable and curative it remains the major health problem in the world, particularly in Sub-Sahara African countries [3]. This is majorly due to the malaria parasites developing resistance to most antimalarial chemotherapies. *For instance, P. falciparum* develops resistance to most all antimalarial including artemisinin-based therapies [1, 4, 5] and *P. vivax* was also found to develop widespread resistance to chloroquine [6–8].

*Eucalyptus globulus (E. globulus*) labill belongs to the Myrtaceae family and the genus *Eucalyptus* which comprises more than 700 species. *E. globulus* is the tallest evergreen tree with its leaves arranged obliquely or vertical shape. It was first discovered by a French explorer in 1792 on the island of Tasmania. *E. globulus* could grow well on a variety of soils and in a variety of climates and is known for its rapid growth [9]. The leaf of *E. globulus* is the main source of essential oil which is widely used in the pharmaceutical and cosmetic industries.

The major chemical composition of *E. globulus* leaf essential oil is monoterpene oxide 1,8-cineole, also known as Eucalyptol which has a wide range of antibiological potentials. In addition, phytochemical screening of the leaf extracts showed the presence of tannins, saponins, terpenoids, glycosides, alkaloids, phenolic compounds, steroids, cardiac glycosides, terpenes, reducing sugars, carbohydrates, resins, acidic compounds, and flavonoids that correlated with positive health influence. Provided this, *E. globulus* is among the commonly used traditional medicinal plants to treat different human ailments including malaria. People in western Cameroon [10], Butembo city (Democratic Republic of Congo) [11], and Ethiopia (22,44) traditionally used different preparation of the leaf of *E. globulus* in the management of malaria.

Several pharmacological activities of the leaf extracts of *E. globulus* were proved in in vivo and/ or in vitro studies. These include but are not excluded; antipyretic [12], analgesic and anti-inflammatory [13], antibacterial activity [14], anthelmintic activity [15], antioxidant [16, 17], lipid peroxidation inhibitory activity [18], have been reported. The result of an in vitro study showed that leaf extract of *E. globulus* had activity against chloroquine resistance *P. falciparum* with IC50 of 26.45 ± 3.32 μg/ml [19]. However, the in vivo antimalarial activities of solvent fractions of the leaf of *E.globulus* didn’t yet study. Thus, the current study aimed to evaluate the in vivo antimalarial activities of solvent fractions of the leaf of *E. globulus.*

## Materials and methods

### Plant material

The fresh leaves of *E. globulus* were collected from Azezo, Central Gondar, Gondar, Ethiopia, on 12 December 2020. The identification and authentication of the leaves were done by Mr. Abiyu Eniyew Molla (a botanist in the Biology Department, University of Gondar) and registered with voucher No. 001MAT/ 2020.

### Extraction and fractionation of plant material

The collected fresh leaf was washed with clean water to remove dusty particles and dried at room temperature. The dried leaf was milled as coarse powder with mortar and pestle. The resulting powder was extracted by cold maceration using 80% methanol with occasional shaking for 72 hours. The macerate was filtered first with gauze cloth followed by Whatman filter paper grade 1. The marc was re-macerated twice to get enough filtrate. The filtrates were concentrated in rotary vapor at 40 °C followed by freezing in a deep freezer at − 20 °C for a night. Further, the water content of the frozen filtrate was removed by lyophilization within a vacuum freeze dryer at − 50 °C. Finally, the extract was packed and stored in a refrigerator until usage.

Chloroform, ethyl acetate, and water were used to fractionate the crude extract in the sequence of increasing polarity. The crude extract was first dissolved in 500 ml of distilled water and transferred to a separatory funnel. Then, 500 ml Of chloroform was added and left for 72 hours. After 72 hours, clear layers of the aqueous and chloroform were formed and the chloroform layer was removed. This was repeated twice with an equal volume of chloroform. Then, the chloroform fractions (CF) were combined and concentrated in a hot air oven at 40 °C. A 500 ml of ethyl acetate was added to the aqueous layer left after chloroform fractionationto get the ethyl acetate fraction (EAF). This was conducted in the same manner as the CF. The aqueous layer which remain after ethylacetate fractionation was lyophilized and used as an aqueous fraction (AF). Finally, the obtained fractionations were transferred into separate vials for storage in the refrigerator until usage.

### Animals

A total of 275 either sex healthy swiss albino mice aged 6 to 8 weeks were used for the experiment. The mice were maintained in the standard condition in a 12-hour light and dark cycle room and fed on standard pellet and water ad libitum. The animals were acclimatized for a week in the experimental room before the experiment. In the experiment, the animals were handled and used in compliance with guidelines of care and use of experimental animals [20]. At the end of the experiment, the infected mice were euthanatized using cervical dislocation.

### Parasite inoculation

Infected mice with chloroquine-sensitive strains of P. berghei ANKA were obtained from Aklilu Lemma Institute of Pathobiology.. Then the parasite was maintained by serial passage of blood from donor mice to noninfected ones on weekly basis. Infected mice with a parasitemia level of 30–37% were used as a donor [21]. The donor mice were anesthetized with ketamine 80 mg/kg IP and the parasitized red blood cells (pRBCs) was collected by cardiac puncture into tube treated with 0.5% trisodium citrate. The collected blood was diluted with normal saline (0.9%) based on the parasitemia level of the donor mice and the RBC count of the normal mice (4.5 × 10^9^ RBC/ml) to obtain the standardized parasitic inoculum with 10^7^ pRBCs in 0.2 ml of suspension. Then the healthy mice were inoculated intraperitoneally with the standardized inoculum.

### Phytochemical screening

Secondary metabolites of the plant are necessary phytochemical constituents for different medicinal uses. Thus**,** the presence of phytochemicals including flavonoids, tannins, alkaloids, cardiac glycosides, saponin, phenol, steroids, and terpenoids were screened out based on the standards [22, 23].

### Acute oral toxicity test

The test was conducted based on Organization for Economic Cooperation and Development (OECD) 425 [24]. For each fraction, healthy five female mice aged 6–8 week were used for the determination of acute oral toxicity. The mice fasted for food but not water for 4 h before the test and 2 h after the test. First, 2000 mg/kg of each fraction was administered through oral gavage to one mouse and monitored continuously for the first 30 min and intermittently for 24 h. Since no death was observed, the same dose of the fractions was administered for the rest four mice. The mice were monitored every 30 min for 4 h and daily for a total of 2 weeks for the general signs and symptoms of toxicity including; changes in skin and fur, patterns of food and water intake, eyes, respiratory behaviors, and mortality.

### Grouping and dosing of animals

The infected mice were randomly divided into five groups each with six mice. Group one (GI), negative control, was treated with 10 ml/kg of 2% tween 80 for chloroform and ethyl acetate fractions and distilled water for an aqueous fraction. Group two (GII), positive control, were treated with chloroquine 25 mg/kg. The rest groups (GIII, GIV, and GV) were treated with 100 mg/kg, 200 mg/kg, and 400 mg/kg of each solvent fractionation.

### Determination of antimalarial activity

#### Four-day suppressive test

The four-day antimalarial activity of *E.globulus* leaf solvent fractions against *P.berghei* was conducted according to Peters 4-day suppressive test [25]. The mice were inoculated intraperitoneally with 0.2 mL of standard inoculum of pRBCs on the first day (D0) of the experiment. Then the mice were grouped and treated as mentioned earlier under the grouping and dosing section. The treatment was begun 2 hours post-inoculation of the parasite and continued on a daily dosage until the fourth day (D3).

#### Curative test (Rane’s test)

The effect of *E.globulu* leaf solvent fractions against already established *P.berghei* infection in mice was evaluated according to the method employed by Ryley and Peters [26]. The mice were inoculated intraperitoneally with 0.2 mL of standard inoculum of pRBCs on the first day (D0) of the experiment and left untreated for 72 hours (D2). Then the mice were grouped and treated as mentioned earlier. The treatment was begin on the fourth day (D3) and continued daily until the seventh day (D6) of the experiment.

#### Prophylactic (repository test)

The prophylactic activity of *E.globulus* leaf solvent fractions was tested based on the method applied by Peters [27]. The mice were grouped as mentioned previously and treated according to. The mice were treated once per day for 4 days (D0-D3). On the 5th day (D4) of the experiment, the mice were inoculated with 0.2 ml standardized pRBCs intraperitoneally and followed until the seventh (D6).

#### Determination of parasitemia level and parasitemia suppression

Blood was collected from the tail vein of each mouse on D4 in four–day suppressive tests and D7 in curative test and repository test. A thin smear of the blood was made on a microscope slide, fixed with methanol, and stained with 10% Giemsa to determine parasitemia level. The percentage parasitemia and parasitemia suppression were calculated using the following formulas [28].

$$\%\;paracetemia=\frac{Total\ number\ of\ pRBCs\kern0.75em }{Total\ number\ of\ RBCs}x\ 100$$

$$\%\;parasitemia\ suppression=\frac{\%\;Parasitemia\ \left( control\ group-\kern0.5em study\ group\right)}{\ Parasitemia\ in\ the\ control\ group\ }\ x100$$

#### Determination of percent packed cell volume and percent change

The antihemolytic activity of the solvent fractions against parasite-induced hemolysis is determined by measuring packed cell volume (PCV). Blood from the tail vein of each mouse was collected on the days just before and after the treatment using heparinized microhematocrit capillary tubes. The tubes were filled up to 3/4th of their height and sealed well. Then the tubes were placed in a microhematocrit centrifuge with their sealed end up and centrifugated at 1200 rpm for 15 minutes [29]. Then the percent PCV and percent change in PCV were determined using the following formula [29]

$$\%\;PCV=\frac{Volume\ of\ erythrocytes\ in\ a\ given\ volume\ of\ blood}{\kern0.5em Total\ blood\ volume\kern0.75em }x\ 100$$

$$\%\;change\ in\ PCV=\frac{mean\;of\;\%\;PCV\ at\ \left( Da- Db\right)}{Mean\;\ of\;\%\;PCV\kern0.5em at\ Db\kern0.5em }x\ 100$$

*Where; Da = day just before the treatment and Db = day just after the treatment.*

#### Determination of mean survival time

Agents with antimalarial activity could increase the survival time of the infected one. Thus measuring mean survival time (MST) is one of the parameters used to assess the antimalarial activity of solvent fractions. For the three antimalarial test models, the infected mice both the treated and controls were monitored for 30 days, and death was recorded daily. Then the MST was calculated using the following formula.

$$MST=\frac{Total\ survival\ time\ of\ mice\ in\ a\ group}{\ Total\ number\ of\ mice\ in\ a\ group}$$

#### Determination of change in body weight and temperature

Since malaria-infected mice develop loss of body weight and body temperature, recording the change of these parameters is used to evaluate the antidiarrheal activity of the solvent fractions. The body weight and temperature of each mouse were recorded on D0 and D4 in four–day suppressive test, D3 and D7 in the curative test, and D0 and D7 in the repository test. The percent change of body weight and temperature were determined as follows:

$$\%\;change\ of\ body\ weight=\frac{Mean\ body\ weight\ at\ \left( Da- Db\right)\ }{Mean\ body\ weight\ at\kern0.75em Db}x\ 100$$

$$\%\;change\ in\ rectal\ temperature=\frac{Mean\ rectal\ temperature\ at\ \left( Da- Db\right)}{Mean\ rectal\ temperature\ at\ Db}x\ 100$$

*Where; Da = day just after the treatment and Db = Day just before the treatment.*

### Data analysis

Results of the study were expressed as mean ± standard error of the mean. Comparison of means was conducted by using one-way analysis of variance (ANOVA) followed by post hoc Tukey’s test with version 21 SPSS at a 95% confidence interval.

## Results

### Preliminary phytochemical screening

Preliminary phytochemical screening of the leaf fractions of *E. globulus* showed the presence of several metabolites in CF and EAF and few metabolites in the AF (Table 1).

**Table 1:** Preliminary phytochemical constituents of solvent fractions of the leaf of *E .globulus*

Phytochemicals	Chloroform fraction	Ethyl acetate fraction	Aqueous fraction
Alkaloids	+	+	_
Flavonoids	+	+	_
Tannins	+	+	+
Saponins	_	_	_
Terpenoids	+	+	+
Phenols	+	+	+
Cardiac glycosides	_	+	_
Steroids	+	+	+

*+present*

*-absent*

### Acute oral toxicity test

The mice were monitored every 30 minutes for 4 hours and daily for 14 days and observed no behavioral change or death mice. This suggested the LD50 of each fraction was above 2000 mg/kg.

### Four–day suppressive test

In this test model, the CF and EAF at all test doses and the AF at 400 mg/kg suppressed blood parasite level and increased the mean survival time of the mice (*p* < 0.001) compared to negative controls. In contrast, both effects of all solvent fractions were significantly lower (*p* < 0.001) compared to the effect of chloroquine (Table 2). Reduction in body weight and temperature of the mice were prevented (*p* < 0.001) at 400 mg/kg of all fractions compared to the negative controls and had a nearly similar effect to chloroquine (Table 3). Packed cell volume reduction was prevented (*p* < 0.05 to *p* < 0.001) at all test doses of all fractions compared to negative controls. In addition, all fractions at 200 mg/kg and 400 mg/kg had a close effect on chloroquine (Table 4).

**Table 2:** The effects of *E. globulus* leaf solvent fractions against *P. berghei* infected mice in the 4-day suppressive test

Treatments	% Parasitemia	% Parasitemia suppression	Mean survival time (day)
2%tween 80 (10 ml/kg)	34.67 ± 2.16	–	9.50 ± 0.99
CQ (25 mg/kg)	0.00 ± 0.00	100.00 ± 0.00^a3^	29.50 ± 0.34^a3^
CF (mg/kg) 100	17.00 ± 0.89	52.91 ± 3.43^a3b3^	16.83 ± 0.95 ^a3b3^
200	12.33 ± 0.67	66.08 ± 1.58^a3b3^	17.17 ± 0.48^a3b3^
400	11.00 ± 0.93	69.93 ± 2.01^a3b3^	19.00 ± 1.06^a3b3^
EAF (mg/kg) 100	16.00 ± 0.97	53.71 ± 1.64^a3b3^	16.00 ± 0.73^a3b3^
200	13.67 ± 0.71	60.31 ± 1.66^a3b3^	16.17 ± 1.01^a3b3^
400	10.83 ± 0.79	68.13 ± 3.05^a3b3^	22.50 ± 0.92^a3b3^
DW (10 ml/kg)	36.50 ± 1.65	–	9.00 ± 0.73
CQ (25 mg/kg)	0.00 ± 0.00	100.00 ± 0.00^a3^	29.50 ± 0.34^a3^
AF (mg/kg) 100	26.17 ± 1.01	23.28 ± 5.05^a3b3^	10.33 ± 1.02 ^b3^
200	22.33 ± 0.95	34.01 ± 5.52^a3b3^	12.17 ± 0.75^b3^
400	16.83 ± 0.91	50.61 ± 3.83^a3b3^	15.67 ± 0.67^a3b3^

Data are expressed as mean ± SEM

*CF* Chloroform fraction, *EAF* Ethyl acetate fraction, *DW* Distilled water, *AF* Aqueous fraction, *CQ* Chloroquine

^1^*p* < 0.05

^2^*p* < 0.01, *p* < 0.001

^a^compared to negative controls

^b^compared to chloroquine

**Table 3:** The effects of *E. globulus* leaf fractions on body weight and temperature against *P.berghei* infected mice in the 4-day suppressive test

Treatments	Bodyweight at D0 (g)	Bodyweight at D4 (g)	% Change in body weight	The rectal temperature at D0(°C)	The rectal temperature at D4(°C)	% change in rectal temperature
2%tween80 (10 ml/kg)	25.33 ± 1.43	22.58 ± 1.36	−10.90 ± 1.22	35.40 ± 0.58	32.58 ± 0.53	−7.95 ± 0.38
CQ (25 mg/kg)	26.52 ± 0.70	26.88 ± 0.66	1.45 ± 1.51^a3^	35.80 ± 0.51	35.95 ± 0.31	0.48 ± 1.00^**a3**^
CF (mg/kg) 100	26.50 ± 1.09	24.53 ± 1.04	−7.44 ± 0.47^**b2**^	36.48 ± 0.33	34.25 ± 0.57	−6.14 ± 1.10^**b3**^
200	24.17 ± 0.91	22.50 ± 0.83	−6.87 ± 0.92^**b2**^	36.35 ± 0.32	34.82 ± 0.48	−4.23 ± 0.55^**a1b2**^
400	24.68 ± 1.23	24.78 ± 1.24	0.44 ± 1.21^**a3**^	36.45 ± 0.44	36.17 ± 0.31	−0.74 ± 0.77^**a3**^
EAF (mg/kg)100	28.05 ± 0.60	25.85 ± 0.70	−7.90 ± 0.57^**b3**^	36.13 ± 0.49	34.03 ± 0.63	−5.84 ± 0.63^**b3**^
200	25.73 ± 1.44	23.65 ± 1.52	−8.29 ± 1.50^**b3**^	36.02 ± 0.51	35.03 ± 0.43	−2.71 ± 0.50^**a1**^
400	25.77 ± 1.561.01	25.93 ± 1.29	1.07 ± 2.05^**a3**^	36.35 ± 0.55	36.50 ± 0.26	0.48 ± 0.90^**a3**^
DW (10 ml/kg)	26.67 ± 1.23	23.97 ± 1.27	−10.24 ± 1.01	36.03 ± 0.42	33.62 ± 0.54	−6.73 ± 0.48
CQ (25 mg/kg)	26.52 ± 0.70	26.88 ± 0.66	1.45 ± 1.51^**a3**^	35.80 ± 0.51	35.95 ± 0.31	0.48 ± 1.00^a**3**^
AF (mg/kg) 100	26.28 ± 0.88	24.30 ± 0.94	−7.62 ± 0.64^**b2**^	36.17 ± 0.39	34.30 ± 0.39	−5.16 ± 0.52^**b3**^
200	27.42 ± 1.01	25.60 ± 0.91	−6.57 ± 1.00^**b2**^	35.90 ± 0.35	34.78 ± 0.31	−3.09 ± 0.74^**a2b1**^
400	26.25 ± 0.58	26.08 ± 0.82	−0.62 ± 2.34^**a3**^	36.12 ± 0.32	36.17 ± 0.28	0.16 ± 0.72^**a3**^

Data are expressed as mean ± SEM

*CF* Chloroform fraction, *EAF* Ethyl acetate fraction, *DW* Distilled water, *AF* Aqueous fraction, *CQ* Chloroquine

^1^*p* < 0.05

^2^*p* < 0.01, *p* < 0.001

^a^compared to negative controls

^b^compared to chloroquine

**Table 4:** The effect of solvent fractions of the leaf of *E.globulus* on packed cell volume against *P.berghei* infected mice in the 4-day suppressive test

Treatments	Packed cell volume		
	D0	D4	%change
2%tween 80(10 ml/kg)	48.33 ± 2.08	42.67 ± 1.93	−11.75 ± 0.80
CQ (25 mg/kg)	49.00 ± 1.53	49.67 ± 1.71	1.34 ± 1.01^a3^
CF (mg/kg)100	49.17 ± 1.97	48.00 ± 2.21	−2.48 ± 0.70^a3^
200	51.00 ± 2.02	50.83 ± 1.82	−0.22 ± 1.08^a3^
400	50.67 ± 1.82	51.17 ± 1.87	0.99 ± 1.13^a3^
EAF (mg/kg) 100	49.00 ± 1.86	46.67 ± 1.91	−4.82 ± 0.53^a2b1^
200	49.00 ± 2.59	46.67 ± 1.71	−4.16 ± 3.04^a2^
400	49.50 ± 2.59	49.33 ± 2.64	−0.35 ± 0.85^a3^
DW (10 ml/kg)	51.00±	45.50 ± 2.22	−10.87 ± 0.95
CQ (25 mg/kg)	49.00 ± 1.53	49.67 ± 1.71	1.34 ± 1.01^a3^
AF (mg/kg)100	49.50 ± 1.18	46.83 ± 1.25	−5.42 ± 0.47^a1b2^
200	50.67 ± 2.03	49.17 ± 2.12	−3.02 ± 0.51^a3^
400	49.83 ± 2.80	49.17 ± 2.83	−1.38 ± 0.45^a3^

Data are expressed as mean ± SEM.

*CF* Chloroform fraction, *EAF* Ethyl acetate fraction, *DW* Distilled water, *AF* Aqueous fraction, *CQ* Chloroquine

^1^*p* < 0.05

^2^*p* < 0.01, *p* < 0.001

^a^compared to negative controls

^b^compared to chloroquine

#### Rane’s test

In this test, all fractions at three test doses suppressed blood parasite level and increased mean survival time significantly (*p* < 0.001) compared to the negative controls. In contrast, the effect of the fractions against blood parasite level and mean survival time at all test doses were lower (*p* < 0.001) compared to chloroquine (Table 5). Parasite-induced body weight and temperature reduction were prevented (*p* < 0.01 to *p* < 0.001) at 200 mg/kg and 400 mg/kg of CF and EAF, and 400 mg/kg of AF compared to the negative controls, and these showed a close effect to chloroquine (Table 6). The reduction in PCV was significantly lower (p < 0.001) at 200 mg/kg and 400 mg/kg of the three fractions compared to the negative controls (Table 7).

**Table 5:** The effects of *E. globulus* leaf solvent fractions against P. berghei infected mice in Rane’s test

Treatments	% parasitemia (D7)	%parasitemia suppression	Mean survival time (day)
2%tween80 (10 ml/kg)	36.00 ± 1.29	–	8.17 ± 0.70
CQ (25 mg/kg)	0.00 ± 0.00	100.00 ± 0.00^a3^	30.00 ± 0.48^a3^
CF (mg/kg) 100	17.50 ± 0.96	51.17 ± 2.82^a3b3^	15.17 ± 0.65^a3b3^
200	13.17 ± 0.60	63.46 ± 0.78^a3b3^	16.67 ± 0.88^a3b3^
400	11.67 ± 0.71	67.60 ± 1.63^a3b3^	20.83 ± 0.54^a3b3^
EAF (mg/kg) 100	16.33 ± 0.61	54.58 ± 1.07^a3b3^	15.33 ± 0.42^a3b3^
200	14.83 ± 0.60	58.53 ± 2.38^a3b3^	16.83 ± 0.48^a3b3^
400	10.33 ± 0.67	71.11 ± 2.13^a3b3^	22.33 ± 0.71^a3b3^
DW (10 ml/kg)	37.83 ± 1.40	–	7.83 ± 0.48
CQ (25 mg/kg)	0.00 ± 0.00	100.00 ± 0.00^a3^	30.00 ± 0.48^a3^
AF (mg/kg) 100	24.17 ± 0.87	32.15 ± 4.46^a3b3^	11.67 ± 0.71^a3b3^
200	21.33 ± 0.76	40.38 ± 2.80^a3b3^	12.67 ± 0.56^a3b3^
400	15.50 ± 0.43	56.66 ± 1.99^a3b3^	16.67 ± 0.67^a3b3^

Data are expressed as mean ± SEM.

*CF* Chloroform fraction, *EAF* Ethyl acetate fraction, *DW* Distilled water, *AF* Aqueous fraction, *CQ* Chloroquine

^1^*p* < 0.05

^2^*p* < 0.01, *p* < 0.001

^a^compared to negative controls

^b^compared to chloroquine

**Table 6:** Effects of *E. globulus* leaf fractions on body weight and temperature against *P.berghei* infected mice in Rane’s test

Treatments	Bodyweight at D3(g)	Bodyweight at D7 (g)	% Change in body weight	Rectal temperature at D3 (°C)	Rectal temperature at D7 (°C)	% change in rectal temperature
2%tween 80 (10 ml/kg)	24.33 ± 0.71	21.25 ± 0.44	−12.55 ± 1.02	36.07 ± 0.29	33.15 ± 0.42	−8.09 ± 0.80
CQ (25 mg/kg)	25.33 ± 1.17	25.33 ± 0.84	0.34 ± 2.12^**a3**^	35.80 ± 0.51	36.23 ± 0.25	1.27 ± 0.99^**a3**^
CF (mg/kg) 100	27.00 ± 1.13	25.43 ± 1.03	−5.76 ± 0.67	36.48 ± 0.33	34.42 ± 0.20	−5.64 ± 0.65^**b3**^
200	24.33 ± 0.80	23.67 ± 0.99	−2.85 ± 1.35^**a2**^	36.35 ± 0.32	35.50 ± 0.37	−2.34 ± 0.30^**a3**^
400	26.10 ± 0.56	25.83 ± 0.87	−1.12 ± 1.70^**a3**^	36.45 ± 0.44	35.96 ± 0.33	−1.32 ± 0.72^a3^
EAF (mg/kg)100	27.13 ± 0.91	25.27 ± 0.87	−6.88 ± 0.79^**b1**^	36.13 ± 0.49	34.35 ± 0.58	−4.96 ± 0.35^**b3**^
200	25.33 ± 0.92	24.38 ± 1.27	−4.00 ± 1.71^**a2**^	36.02 ± 0.51	35.77 ± 0.31	−0.65 ± 0.80^**a3**^
400	24.33 ± 0.80	25.12 ± 0.73	3.33 ± 1.44^**a3**^	36.35 ± 0.55	36.38 ± 0.28	0.18 ± 1.32^**a3**^
DW (10 ml/kg)	26.67 ± 1.02	24.30 ± 1.12	−8.99 ± 0.93	36.03 ± 0.42	33.18 ± 0.49	−7.92 ± 0.56
CQ (25 mg/kg)	25.33 ± 1.17	25.33 ± 0.84	0.34 ± 2.12^**a3**^	35.80 ± 0.51	36.23 ± 0.25	1.27 ± 0.99^**a3**^
AF (mg/kg) 100	25.67 ± 0.56	24.02 ± 0.79	−6.53 ± 1.17	36.17 ± 0.39	33.85 ± 0.42	−6.41 ± 0.67^**b3**^
200	26.10 ± 1.48	25.32 ± 1.28	−2.77 ± 1.92	35.90 ± 0.35	35.27 ± 0.22	−1.73 ± 1.01^**a3**^
400	27.00 ± 0.37	26.95 ± 0.55	−0.17 ± 1.7**5**^**a2**^	36.12 ± 0.32	35.93 ± 0.31	−0.49 ± 0.88^**a3**^

Data are expressed as mean ± SEM.

*CF* Chloroform fraction, *EAF* Ethyl acetate fraction, *DW* Distilled water, *AF* Aqueous fraction, *CQ* Chloroquine

^1^*p* < 0.05

^2^*p* < 0.01, *p* < 0.001

^a^compared to negative controls

^b^compared to chloroquine

**Table 7:** The effect of solvent fractions of *E.globulus* on packed cell volume against *P.berghei* infected mice in Rane’s test

Treatments	Packed cell volume		
	D3	D7	% change
2% tween80(10 ml/kg)	45.67 ± 0.84	41.00 ± 1.21	−10.31 ± 1.12
CQ (25 mg/kg)	44.17 ± 1.05	44.33 ± 0.95	0.42 ± 0.70^a3^
CF (mg/kg) 100	44.50 ± 1.23	43.00 ± 1.37	−3.42 ± 0.56^a3^
200	44.00 ± 1.44	44.00 ± 0.97	0.23 ± 1.70^a3^
400	42.83 ± 1.01	43.17 ± 0.65	0.92 ± 1.48^a3^
EAF (mg/kg)100	45.00 ± 1.39	43.17 ± 1.49	−4.12 ± 0.75^a2^
200	45.17 ± 0.95	44.33 ± 0.84	−1.82 ± 0.65^a3^
400	43.33 ± 1.48	44.00 ± 1.03	1.75 ± 1.60^a3^
DW (10 ml/kg)	45.67 ± 1.26	40.50 ± 1.26	−11.36 ± 0.46
CQ (25 mg/kg)	44.17 ± 1.05	44.33 ± 0.95	0.42 ± 0.70^a3^
AF (mg/kg) 100	46.00 ± 1.10	42.83 ± 1.30	−6.95 ± 0.78^a1b3^
200	46.33 ± 0.71	45.00 ± 0.86	−2.90 ± 0.49^a3^
400	50.17 ± 2.57	49.50 ± 2.54	−1.31 ± 0.90^a3^

Data are expressed as mean ± SEM.

*CF* Chloroform fraction, *EAF* Ethyl acetate fraction, *DW* Distilled water, *AF* Aqueous fraction, *CQ* Chloroquine

^1^*p* < 0.05

^2^*p* < 0.01, *p* < 0.001

^a^compared to negative controls

^b^compared to chloroquine

#### Prophylactic test

Blood parasite level was suppressed significantly (*p* < 0.001) at all test doses of the fractions compared to the negative controls. The mean survival time of the mice was also increased (*p* < 0.05 to *p* < 0.001) compared to the negative controls. In contrast, the percentage parasitemia suppression and increase in mean survival time within the fractions at all test doses were significantly lower compared to chloroquine (Table 8). The loss of body weight and temperature were significantly reduced (*p* < 0.01 to *p* < 0.001) at 200 mg/ kg and 400 mg/kg of the fractions compared to negative controls and these showed the closest effects to chloroquine (Table 9). The reduction in packed cell volume was prevented (*p* < 0.001) at all test doses of the fractions compared to the negative controls. In addition, 200 mg/kg and 400 mg/kg of all fractions showed the close effect of chloroquine in the prevention of packed cell volume reduction (Table 10).

**Table 8:** Chemo-prophylactic activity of *E. globulus* leaf solvent fractions against *P. berghei* infected mice

Treatments	% parasitemia (D7)	%parasitemia suppression	Mean survival time (day)
2% tween 80 (10 ml/kg)	36.67 ± 1.28	0.00 ± 0.00	9.67 ± 0.80
CQ (25 mg/kg)	5.50 ± 0.56	85.10 ± 1.19	28.33 ± 0.49^a3^
CF (mg/kg) 100	19.17 ± 1.22	48.19 ± 4.43^a3b3^	14.33 ± 0.80^a2b3^
200	14.83 ± 0.60	60.15 ± 1.75^a3b3^	17.67 ± 0.71^a3b3^
400	13.67 ± 0.71	63.29 ± 0.71^a3b3^	19.50 ± 0.71^a3b3^
EAF (mg/kg) 100	16.33 ± 0.71	55.27 ± 2.13^a3b3^	15.67 ± 0.67^a2b3^
200	16.00 ± 0.37	55.27 ± 2.13^a3b3^	16.83 ± 0.60^a3b3^
400	12.67 ± 0.61	65.20 ± 2.20^a3b2^	20.17 ± 0.70^a3b3^
DW (10 ml/kg)	37.33 ± 1.12	0.00 ± 0.00	9.17 ± 0.40
CQ (25 mg/kg)	5.50 ± 0.56	85.34 ± 1.28^a3^	28.33 ± 0.49^a3^
AF (mg/kg) 100	24.33 ± 0.88	33.14 ± 3.54^a3b3^	11.67 ± 1.20^b3^
200	21.67 ± 0.49	40.53 ± 2.65^a3b3^	13.83 ± 0.48^b3^
400	15.67 ± 0.88	57.13 ± 2.48^a3b3^	16.00 ± 1.06^a3b3^

Data are expressed as mean ± SEM.

*CF* Chloroform fraction, *EAF* Ethyl acetate fraction, *DW* Distilled water, *AF* Aqueous fraction, *CQ* Chloroquine.

^1^*p* < 0.05

^2^*p* < 0.01, *p* < 0.001

^a^compared to negative controls

^b^compared to chloroquine

**Table 9:** Effects of *E. globulus* leaf fractions on body weight and temperature against *P. berghei* infected mice in the prophylactic test

Treatments	Bodyweight at D0 (g)	Bodyweight at D7 (g)	% change in body weight	Rectal temperature at D0 (°C)	Rectal temperature at D7 (°C)	% change in rectal temperature
2%tween80(10 ml/kg)	24.83 ± 0.60	22.42 ± 0.55	−9.72 ± 0.76	35.33 ± 0.33	32.67 ± 0.46	−7.55 ± 0.88
CQ(25 mg/kg)	25.67 ± 0.76	26.47 ± 0.56	3.28 ± 1.43^a3^	35.85 ± 0.36	35.95 ± 0.31	0.30 ± 0.83
CF (mg/kg) 100	26.17 ± 1.19	25.18 ± 1.06	−3.66 ± 0.81^a1b2^	36.63 ± 0.33	35.33 ± 0.44	−3.55 ± 0.88
200	24.33 ± 0.95	23.75 ± 0.81	−2.26 ± 1.28^a2b1^	35.60 ± 0.46	35.32 ± 0.47	−0.74 ± 1.55^a3^
400	24.33 ± 1.17	24.53 ± 1.32	0.72 ± 1.40^a3^	36.40 ± 0.51	36.33 ± 0.33	−0.12 ± 1.29^a3^
EAF (mg/kg) 100	26.17 ± 0.91	5.00 ± 0.85	−4.44 ± 0.31^b2^	35.58 ± 0.37	34.70 ± 0.40	−2.46 ± 1.09^a1^
200	25.93 ± 1.26	25.22 ± 0.95	−2.53 ± 1.11^a2b1^	35.92 ± 0.49	35.52 ± 0.43	−1.10 ± 0.54^a3^
400	23.17 ± 1.08	24.02 ± 1.02	3.82 ± 1.49^a3^	36.83 ± 0.38	36.92 ± 0.08	0.28 ± 0.97^a3^
DW (10 ml/kg)	27.67 ± 0.71	25.13 ± 0.64	−9.15 ± 0.50	36.27 ± 0.35	33.62 ± 0.44	−7.32 ± 0.64
CQ (25 mg/kg)	25.67 ± 0.76	26.47 ± 0.56	3.28 ± 1.43^a3^	35.85 ± 0.36	35.95 ± 0.31	0.30 ± 0.83^a3^
AF (mg/kg) 100	25.67 ± 0.88	24.33 ± 0.99	−5.28 ± 1.13^b3^	35.88 ± 0.47	34.43 ± 0.45	−4.03 ± 0.65
200	28.42 ± 1.07	27.70 ± 0.83	−2.36 ± 1.19^a2b1^	35.57 ± 0.39	35.05 ± 0.28	−1.44 ± 0.40^a2^
400	25.93 ± 0.65	26.00 ± 0.43	0.40 ± 1.47^a3^	36.38 ± 0.28	36.25 ± 0.28	−0.36 ± 0.66^a3^

Data are expressed as mean ± SEM.

*CF* Chloroform fraction, *EAF* Ethyl acetate fraction, *DW* Distilled water, *AF* Aqueous fraction, *CQ* Chloroquine

^1^*p* < 0.05

^2^*p* < 0.01, *p* < 0.001

^a^compared to negative controls

^b^compared to chloroquine

**Table 10:** The effect of solvent fractions of the leaf of *E.globulus* on packed cell volume against *P.berghei* infected mice in the prophylactic test

Treatments	Packed cell volume		
	D0	D7	% change
2%tween80 (10 ml/kg)	48.50 ± 1.57	43.50 ± 1.80	−10.42 ± 1.26
CQ (25 mg/kg)	48.33 ± 1.84	48.33 ± 1.69	0.08 ± 0.76^a3^
CF (mg/kg) 100	46.00 ± 2.03	44.33 ± 1.99	−3.64 ± 0.47^a3^
200	47.33 ± 1.45	47.00 ± 1.48	−0.70 ± 0.74^a3^
400	47.17 ± 1.58	47.17 ± 1.25	0.14 ± 1.14^a3^
EAF (mg/kg) 100	47.67 ± 1.89	45.67 ± 1.74	−4.17 ± 0.47^a3b1^
200	47.33 ± 1.28	47.00 ± 1.44	−0.73 ± 1.02^a3^
400	48.17 ± 1.82	48.00 ± 1.93	−0.37 ± 0.95^a3^
DW (10 ml/kg)	46.33 ± 0.84	41.33 ± 0.88	−10.81 ± 0.59
CQ (25 mg/kg)	48.33 ± 1.84	48.33 ± 1.69	0.08 ± 0.76
AF (mg/kg) 100	46.17 ± 1.47	44.00 ± 1.32	−4.66 ± 0.61^a3b2^
200	46.50 ± 0.62	45.50 ± 0.43	−2.11 ± 0.77^a3^
400	46.33 ± 1.41	46.17 ± 1.40	−0.35 ± 0.68^a3^

Data are expressed as mean ± SEM

*CF* Chloroform fraction, *EAF* Ethyl acetate fraction, *DW* Distilled water, *AF* Aqueous fraction, *CQ* Chloroquine

^1^*p* < 0.05

^2^*p* < 0.01, *p* < 0.001

^a^compared to negative controls

^b^compared to chloroquine

## Discussion

The acute oral toxicity test result showed that the solvent fractions are safe. Provided this, the 4- day suppressive, curative, and prophylactic activity of solvent fractions against *P. berghei* infected mice were done. In contrast to the in vitro activity test, the in vivo antimalarial activity test of the fractions enables to account for the prodrug effect of the fractions and the involvement of the host immune system. *P. berghei* which is sensitive to chloroquine is the commonly used rodent malaria parasite to investigate the antimalarial activity of the plant extract in vivo [30]. Because of this, chloroquine was used as a standard drug against *P. berghei*-infected mice in the current study.

Compounds that showed percentage parasitemia suppression above or equal to 30% were considered as active against malaria infection [31]. In agreement with this, all three doses of CF and EAF and the middle and large dose of AF in the 4-day suppressive test, and all fractions at all test doses in curative and prophylactic tests showed percentage parasitemia suppression above 30%. If the plant extract showed 50% or above in vivo parasitemia suppression, the effect could be classified as moderate, good, and very good at 500 mg, 250 mg, and 100 mg/kg, respectively [32]. In line with this, the CF and EA at all three test doses in three antimalarial test models showed above 50% parasitemia suppression. Thus CF and EAF had very good antimalarial activity. In contrast, the AF showed 50% and more parasitemia suppression only at 400 mg/kg. This might be attributed to the absence or low concentration of active secondary metabolites in the middle and lower test doses of the AF. In addition, these fractions improved the mean survival time of the infected mice owing to significant suppression of the parasitemia level. The plant extract with antimalarial activity could prolong the survival time of the infected mice for more than 12 days [33]. In compliance with this, all fractions at all test doses in all test models prolonged the mean survival time of the mice greater than 12 days, except the AF at a lower dose.

Rodent malarial infection is commonly manifested by a reduction in body weight, rectal temperature, and PCV. Thus, a plant extract with antimalarial activity could ameliorate the loss of body weight and rectal temperature and reduction of PCV [34, 35]. In the 4-day suppressive test, the CF and EAF at 400 mg/kg produced an increment in body weight. In addition, CF in the prophylactic test and the EAF in Rane’s test, both at 400 mg/kg showed an increment in body weight. This test model-related effect difference could be due to quantitative and qualitative metabolite constituent variability in each fraction and their pharmacokinetic and pharmacodynamics properties. Furthermore, the CF and EAF at 400 mg/kg in the 4-day suppressive test and 400 mg/kg of EAF in Rane’s test showed an increment in body temperature.

An increase in parasitemia level can cause destructions (hemolysis) of infected and non-infected RBCs, erythropoietin suppression, and dyserythropoietic. These are involved both in human and rodent anemia and decrease PCV [36, 37]. Thus, a plant extract with antimalarial activity could prevent hemolysis and decrease PCV. Regarding this, the CF and EAF at all test doses and models prevented the reduction of PCV compared to the negative controls. The AF at all test doses in a 4-day suppressive test and 200 mg/kg and 400 mg/kg in Rane’s test prevented the decrease in PCV compared to negative controls. These preventive activities of the fractions may be due to the removal of parasites from infected RBCs before hemolysis, improving erythropoiesis, decreasing invasion of non-infected RBCs, and decreasing intraerythrocytic development [37, 38].

The antimalarial activity of plant extracts relies on the presence of different secondary metabolites. These include alkaloids, flavonoids, terpenoids, steroids, and other metabolites [31, 39]. In this regard, the lower antimalarial activity of the AF observed in this study could be because of the presence of few metabolites. The antimalarial activity of the metabolites is induced through different mechanisms. These include preventing RBC invasion, inhibiting the growth and multiplication of the parasite, blocking the entry of essential nutrients into the parasite, cytotoxic to the parasite, inhibiting heme polymerization, and boosting the host immune system [40–42].

Malarial infection is characterized by increasing the release of free radicals and inflammatory mediators like; cytokines and prostaglandins which potentiate the pathogenic effect of the parasite [43]. Thus in addition to the above-mentioned antimalarial mechanisms of metabolites, the antioxidant [16, 17] and anti-inflammatory [13] activities of the *E. globulus* could contribute to ameliorating the pathogenic process of the parasite.

## Conclusion

The result of this study could be concluded as all fractions had potential antimalarial activity. Particularly, the CF and EAF had very good antimalarial activity at all test doses. The AF had very good antimalarial activity only at 400 mg/kg. This might be related to the presence of a few active metabolites in the AF. Indeed, the results of the current study strengthen the observed in vitro antimalarial activity of the hydro methanol extract of leaf of *E. globulus* and its use in traditional medicine to manage malaria*.* This could be used by the scientific community to investigate further the specific active compound and its respective mode of action that leads to the development of the new and effective antimalarial drug.

## Data Availability

All the necessary data supporting the result and conclusion of the study have been incorporated in the manuscript.

## Abbreviations

AF: Aqueous fraction
CF: Chloroform fraction
EAF: Ethyl acetate fraction
PCV: Packed cell volume
